# Cannabidiol Treatment Might Promote Resilience to Cocaine and Methamphetamine Use Disorders: A Review of Possible Mechanisms

**DOI:** 10.3390/molecules24142583

**Published:** 2019-07-16

**Authors:** Claudia Calpe-López, M. Pilar García-Pardo, Maria A. Aguilar

**Affiliations:** 1 Unit of Research “Neurobehavioural mechanisms and endophenotypes of addictive behavior”, Department of Psychobiology, University of Valencia, Avda. Blasco Ibañez 21, 46010 Valencia, Spain; 2 Department of Psychology and Sociology, University of Zaragoza, 44003 Teruel, Spain

**Keywords:** cannabidiol, cocaine, methamphetamine, mice, rat, reinstatement, reward, self-administration, conditioned place preference, addiction

## Abstract

Currently, there are no approved pharmacotherapies for addiction to cocaine and other psychostimulant drugs. Several studies have proposed that cannabidiol (CBD) could be a promising treatment for substance use disorders. In the present work, the authors describe the scarce preclinical and human research about the actions of CBD on the effects of stimulant drugs, mainly cocaine and methamphetamine (METH). Additionally, the possible mechanisms underlying the therapeutic potential of CBD on stimulant use disorders are reviewed. CBD has reversed toxicity and seizures induced by cocaine, behavioural sensitization induced by amphetamines, motivation to self-administer cocaine and METH, context- and stress-induced reinstatement of cocaine and priming-induced reinstatement of METH seeking behaviours. CBD also potentiated the extinction of cocaine- and amphetamine-induced conditioned place preference (CPP), impaired the reconsolidation of cocaine CPP and prevented priming-induced reinstatement of METH CPP. Observational studies suggest that CBD may reduce problems related with crack-cocaine addiction, such as withdrawal symptoms, craving, impulsivity and paranoia (Fischer et al., 2015). The potential mechanisms involved in the protective effects of CBD on addiction to psychostimulant drugs include the prevention of drug-induced neuroadaptations (neurotransmitter and intracellular signalling pathways changes), the erasure of aberrant drug-memories, the reversion of cognitive deficits induced by psychostimulant drugs and the alleviation of mental disorders comorbid with psychostimulant abuse. Further, preclinical studies and future clinical trials are necessary to fully evaluate the potential of CBD as an intervention for cocaine and methamphetamine addictive disorders.

## 1. Introduction

Cocaine and methamphetamine (METH) are addictive psychostimulant drugs that are commonly abused and cause significant morbidity and mortality [1,2]. Abuse of these drugs induces severe physical consequences (seizures, ischemic strokes, myocardial infarction, and acute liver injury) and mental health disorders (anxiety, mood disturbances, cognitive impairments, psychotic symptomatology). For these reasons, the use of these psychostimulant drugs is currently a public health problem in developed countries over the world. Moreover, there are no approved pharmacotherapies for the treatment of cocaine and METH use disorders. Numerous pharmacological compounds are being investigated in preclinical models and clinical trials, but to this date the results are largely disappointing [3,4,5].

Cannabidiol (CBD) is the second most abundant phytocannabinoid component of the cannabis plant, behind delta-9-tetrahydrocannabinol (THC) [6,7,8,9]. However, unlike THC, CBD has low affinity for the cannabinoid receptors CB1 and CB2 [10]. CBD’s mechanism of action is not fully understood yet. Despite CBD not having intrinsic efficacy on CB1 receptors, it acts as a non-competitive antagonist [11,12] and exerts a negative allosteric modulation on these receptors [12,13]. On CB2 receptors, CBD acts as a partial agonist [13]. Furthermore, CBD has an inhibitory action on anandamide uptake and on the fatty acid amide hydroxylase (FAAH), the main endocannabinoid enzyme that metabolises anandamide, augmenting the endocannabinoid tone [14,15,16]. CBD is also an antagonist of the CB1/CB2 agonists [9,11]. Recent studies suggest that CBD inhibits the endocannabinoid system, probably modifying the activity of endocannabinoids (and other primary ligands, such as THC) [13,17]. This ability of CBD to modulate the endocannabinoid signalling pathway can explain why this compound may decrease the activity of CB1 receptors without inducing side effects [18,19]. 

CBD also acts on a number of receptors of different neurotransmitters systems. CBD has agonistic properties at 5-HT1A receptors [20,21]. On the other hand, CBD dose-dependently reverses 5-HT-evoked currents and decreases the efficacy of 5-HT, although it does not alter its potency or compete with the 5-HT3 receptor binding site [22,23]. These results support the idea that CBD acts as an allosteric modulator of 5-HT receptors [10]. Similarly, CBD also induces a non-competitive inhibition of the α7-nicotinic receptors [24], an allosteric modulation of mu- and delta-opioid receptors [25] and a partial agonism of dopamine (DA) D2 receptors [26]. Furthermore, CBD is an agonist at the transient receptor potential A1 (TRPA1), TRPV1 and TRPV2 [27,28,29] and activates the glycine receptors [30]. CBD is also a functional antagonist of the G protein-coupled receptor (GPR) 55 [31] and an inverse agonist for GPR3, GPR6, and GPR12 [32,33].

CBD generally only induces mild side effects in preclinical and clinical studies [18,34,35] and is devoid of motivational properties, suggesting a limited abuse liability [36,37,38,39]. This pharmacological profile has increased the pharmaceutical interest in this compound in recent years. A broad spectrum of pharmacological actions of CBD has been reported, including the modulation of cardiovascular, immune and neuronal function [19]. Furthermore, there is an ever increasing consumer demand for CBD. An anonymous online survey to CBD users (*n* = 2409) recruited through social media showed that almost 62% of them reported using this drug to treat a medical condition, mainly pain, anxiety, depression and sleep disorders [40]. 

In European countries, Canada and New Zealand, a whole cannabis extract containing an approximate 1:1 ratio of THC to CBD (Sativex) was approved for pain and spasticity associated with multiple sclerosis. In the US, CBD was generally considered a Schedule I controlled substance by the US Drug Enforcement Administration until June 2018, when a CBD purified extract from the Cannabis plant (Epidiolex) was approved for the treatment of seizures associated with two rare and severe forms of epilepsy (Lennox-Gastaut and Dravet syndromes) in children. The antioxidant, anti-inflammatory, neuroprotective, immunosuppressive, anticonvulsant and antiemetic effects of CBD, along with its limited side effects, makes it a good therapeutic candidate for a broad variety of diseases, in particular, cancer and neuropsychiatric disorders [19], including epilepsy, schizophrenia, social phobia, post-traumatic stress, depression, bipolar disorder, sleep disorders and Parkinson [41]. Recently, CBD has been proposed as a promising treatment for substance use disorders. Preliminary evidence shows that CBD may have efficacy in treating cannabis, opioid, alcohol, cocaine and nicotine dependence [41,42,43,44,45,46]. However, with respect to psychostimulant substances, the evidence is more limited than with other drugs of abuse and mixed results have been observed. A bibliographic search in the Pubmed database with the keywords, cocaine and cannabidiol, or methamphetamine and cannabidiol, identifies 36 and 12 papers, respectively. After the evaluation of their contents, it can be concluded that only a very low number of these papers studied the influence of cannabidiol treatment on the effects of cocaine or METH (12 and 3 papers, respectively). Two thirds of the studies on the potential therapeutic benefits of CBD to treat psychostimulant addiction were published between 2015 and 2019, indicating that, currently, this is a very productive area of research. In the present work, the existing literature on this topic is reviewed to establish whether there is evidence for the possible usefulness of CBD in the treatment of psychostimulant drug use disorders, with a focus on cocaine and METH. A second objective is to hypothesize the neurobiological substrates or mechanisms involved in these effects of CBD.

## 2. Studies About the Influence of CBD on the Effects of Psychostimulant Drugs

### 2.1. Preclinical Studies

#### 2.1.1. Pharmacokinetic Interactions and Cocaine-Induced Toxicity

The first studies on the interactions between CBD and psychostimulant drugs were initiated in the decade of the 1990s and were aimed to determine how CBD modifies the pharmacokinetics and toxicity of cocaine and other drugs of abuse [47,48,49].

Cocaine is metabolized by nonspecific plasma and tissue esterases. Serum cholinesterase hydrolyzed cocaine to inactive products but the hepatic cytochrome P450 (CYP) enzymes (microsomal oxidative metabolism) are responsible for cocaine N-demethylation, which results in norcocaine, a hepatotoxic metabolite of cocaine [50]. In in vitro experiments, CBD inhibited the activity of the enzymes CYP3A and cocaine N-demethylase in human and mouse liver microsomes [47]. In addition, treatment of mice with CBD reduced the levels of the toxic metabolite nor-cocaine (by decreasing the activity of the enzymes CYP2C, CYP3A and cocaine N-demethylase) and protected mice from hepatotoxicity [47,48].

On the other hand, mice treated with CBD (30 mg/kg) 30–60 min before cocaine administration showed higher levels (2- to 4-fold) of cocaine in the brain and blood, this increase in cocaine levels being accompanied by a potentiation of the pharmacological responses to this drug (higher hyperactivity after cocaine administration in CBD-treated mice). Conversely, pretreatment of mice with CBD had no effect on the brain levels of methylenedioxyphenyl-methamphetamine. According to the authors of that study, these findings provide “a biochemical basis for the common practice of using marijuana concurrently with several drugs of abuse” [49].

More recent studies also support the idea that CBD may have protective actions on the toxic effects of cocaine in the liver and brain. CBD (30 mg/kg) reduced acute liver inflammation and damage induced by cocaine in mice [51]. Furthermore, in a mouse model of cocaine intoxication, the pre-treatment with CBD (30 mg/kg) protected against seizures induced by cocaine, increasing the latency and reducing the duration of seizures [51,52]. 

Taken together, these findings support the idea that CBD might have a clinical application in the treatment of cocaine abuse. 

#### 2.1.2. Motor Activity and Behavioural Sensitisation

The effects of CBD on the motor stimulant properties of cocaine have been scarcely studied. Gerdeman et al. [53] reported that systemic pretreatment of a 1:1 mixture of THC and CBD (or THC alone) did not prevent the induction or expression of locomotor sensitization elicited by daily administration of cocaine in mice. Similarly, a recent study reported that systemic CBD (20 mg/kg) did not affect the acquisition or expression of the behavioural sensitisation induced by cocaine in mice [54]. CBD (2.5 mg/kg) also did not prevent the expression of ethanol-induced behavioural sensitisation [55]. On the contrary, administration of CBD (100 ng/0.5 µL into the nucleus accumbens (NAcc) reversed the hyperactivity, rearing, stereotypies and behavioural sensitisation induced by amphetamine [56]. These contradictory findings in the effects of CBD on behavioural sensitization may be related to the differences between systemic and intra-accumbens administration. Further studies are needed to clarify the effects of CBD on the motor actions of psychostimulant drugs.

#### 2.1.3. Intracranial Self-Stimulation (ICSS) Paradigm

CBD itself did not exhibit reinforcing properties in the ICSS paradigm in rats trained to respond for electrical brain stimulation of the medial forebrain bundle, supporting the low liability of abuse of this compound. This study also demonstrated that CBD (5 mg/kg) did not modify the reward-facilitating effect of cocaine, although this dose of CBD was effective to inhibit the effects of morphine on ICSS [36]. These divergent effects of CBD on cocaine and morphine reward suggest that CBD could be more effective in reducing the hedonic effects of opioid than those of psychostimulant drugs.

#### 2.1.4. Self-Administration Paradigm

The effects of CBD on cocaine self-administration have been evaluated only in two studies. In rats, CBD (5 and 10 mg/kg) did not attenuate cocaine self-administration under a progressive ratio schedule of reinforcement when tested for 30 min and 24 h after treatment [57]. In mice, CBD (20 mg/kg) reduced voluntary consumption of cocaine and the breaking point in a progressive ratio [54]. These apparent opposite results probably indicated that high doses of CBD are needed to reduce cocaine self-administration. In support of this idea, motivation of rats to self-administer METH in a progressive ratio reinforcement schedule was also reduced by a high dose of CBD (80 mg/kg), but not by lower doses (20 and 40 mg/kg) [58]. In addition, the effectiveness of CBD to reduce the reinforcing properties of cocaine in the self-administration paradigm apparently contrasts with its lack of effect in the ICSS, but again this apparent discrepancy may be due to the fact that a low dose of CBD was employed to test its effects on cocaine reward in the ICSS paradigm. Further studies are required to determine the effective doses of CBD to prevent the hedonic effects of cocaine and METH.

Relapse into drug use after periods of abstinence is a core characteristic of addiction and the main cause of concern in the treatment of this disorder. In experimental animals, relapse can be modelled by the reinstatement of drug seeking behaviour after extinction of self-administration. As with human relapse, reinstatement is observed when experimental animals are re-exposed to the drug, to environmental cues associated with drug intake or to a stressful condition (known as priming-, cue- or stress-induced reinstatement, respectively). 

The effects of CBD on reinstatement of cocaine self-administration are controversial. An acute treatment with CBD (5 or 10 mg/kg) did not prevent cue-induced reinstatement in rats after a withdrawal period of 14 days [57]. The treatment with CBD during the 10 days of acquisition of cocaine self-administration also did not affect priming-induced reinstatement of cocaine seeking in mice [54]. Conversely, a transdermal preparation of CBD (15 mg/kg at 24 h intervals for 7 days) attenuated context- and stress-induced reinstatement of cocaine seeking without inducing tolerance, sedative effects, and without interfering with normal motivated behavior [59]. Seemingly, such divergent results could be due to a pharmacokinetic/transport-related difference but, in the authors’ opinion, the dose and schedule of treatment (acute versus chronic) of CBD may be responsible for the opposite results observed between the studies. It is important to note that following the cessation of treatment with CBD, reinstatement remained attenuated for a long time (≈5 months) despite plasma and brain CBD levels remaining detectable only for 3 days [59]. CBD administration (80 mg/kg, but not with lower doses) attenuated priming-induced reinstatement of METH self-administration following extinction [58].

#### 2.1.5. Conditioned Place Preference (CPP) Paradigm

CBD itself does not have motivational properties in the CPP paradigm [38,39,54], supporting its low liability of abuse. Several works have studied the effects of CBD on the acquisition, expression, extinction and reactivation of CPP induced by psychostimulant drugs. In an early study, CBD (5 mg/kg) did not affect the acquisition or expression of amphetamine-induced CPP in rats [38]. In contrast, CBD attenuated the acquisition of cocaine-induced CPP in mice, although this effect was only observed at intermediate doses (10 and 20 mg/kg) while lower or higher doses (5 or 30 mg/kg) were ineffective [54]. In addition, the acute administration of CBD (5 mg/kg) 30 min prior to an extinction trial (consisting of a confinement to the drug-paired floor for 15 min) potentiated the extinction of cocaine- and amphetamine-induced CPP in rats. This effect was also observed with THC and it was not modified by the CB1 receptor antagonist SR141716 [38]. One week after the acquisition of cocaine-induced CPP, the acute administration of CBD (10 mg/kg) immediately after the reactivation of CPP (by confining the rats to the drug-paired chamber for 10 min) impaired the reconsolidation of the cocaine-induced CPP [60]. 

The CPP paradigm has also been used as a model to study the relapse into drug seeking behaviour. After the extinction of a previously learned CPP, exposure to a drug priming dose or to a stressful event can induce the reinstatement of CPP (priming- or stress-induced reinstatement, respectively). CBD (10 μg/5 μL, icv) suppressed priming-induced reinstatement of METH CPP [61]. This preventive effect of CBD on reinstatement was observed even in rats exposed to a deprivation of paradoxical sleep for 24 hours, a stressful condition that facilitates priming-induced reinstatement of METH CPP [61]. 

Taken together, the results obtained with the CPP paradigm indicate that CBD modifies different aspects of the reward induced by psychostimulant drugs. However, to date, no studies have been published on the effects of CBD on reinstatement of cocaine CPP. In our laboratory, the authors are currently performing experiments to evaluate the effects of CBD in priming- and stress-induced reinstatement of cocaine CPP in mice.

### 2.2. Human Studies 

#### 2.2.1. Observational Studies

Observational studies have documented that some crack-cocaine addicts reported an intentional use of cannabis as a form of self-medication to reduce different problems related with crack-cocaine addiction, such as withdrawal symptoms, craving, impulsivity and paranoia [62]. A recent prospective longitudinal study, performed in Canada between 2012 and 2015, with 122 participants who reported using cannabis to reduce crack use, concluded that “a period of intentional cannabis use to reduce crack use was associated with decreased frequency of crack use in subsequent periods” [63]. However, in a pilot study with 28 cocaine-dependent subjects with at least 72 h of abstinence, no differences in the craving induced by a video containing drug cues were observed between cocaine-dependent subjects who also abused or were dependent on cannabis (*n* = 16) and those who only presented cocaine dependence [64]. The results from these studies highlight the need for further research to determine whether cannabis, or some of its components, CBD in particular as it is devoid of addictive potential, might reduce craving and consumption of cocaine.

#### 2.2.2. Clinical Trials

A search in ClinicalTrials.gov and in the EU Clinical Trials Register using the keywords, cannabidiol and cocaine or cannabidiol and methamphetamine, only found one clinical trial about the therapeutic potential of CBD to treat dependence to these drugs of abuse. This double-blind, randomized, controlled, phase-II clinical trial (Cannabidiol and Cocaine Craving/Dependence, ClinicalTrials.gov Identifier: NCT02559167), sponsored by Dr. Jutras-Aswad from the University of Montreal (Canada), is now recruiting participants. An estimated number of 110 current cocaine-dependent subjects are expected to receive placebo or 800 mg of CBD for 92 consecutive days (starting on Day 2 of a 10-day inpatient detoxification period followed by 12 weeks of outpatient follow-up) to evaluate the effects of this compound on cue- and stress-induced cocaine craving (on Day 8 of detoxification) and cocaine use relapse (from 10 to 92 days). Furthermore, at different time-frames other outcome measures are expected to be evaluated, including cocaine withdrawal symptoms, sustained abstinence, addiction severity, anxiety, positive and negative affect, depressive symptoms, memory, attention, impulsivity, decision-making and physiological measures (blood pressure and heart rate; cortisol, anandamide and CBD levels; and inflammatory markers). The first results are expected for September 2019 and the estimated study completion date is December 2019. Until this moment, there is no evidence of the effects of CBD in humans with psychostimulant drugs-use related disorders.

## 3. Possible Mechanisms Involved in the Protective Effects of CBD on Addiction to Psychostimulant Drugs

Cocaine and METH use disorders are chronically relapsing conditions. Even after successful detoxification and long-term abstinence, there is a high risk of relapse. Multiple factors can contribute to the enhanced relapse vulnerability in drug addicts, including drug-induced alterations as well as environmental and individual variables. Chronic exposure to cocaine and METH induces alterations on brain structures, neurotransmitter systems and molecular pathways. Cues or contexts associated with drug consumption and stress exposure are the main environmental contributors to relapse. These environmental factors trigger craving and anxiety increasing the probability of drug use relapse, although there are differences in the susceptibility of subjects to these environmental challenges. Cognitive dysfunctions, impaired impulse control and co-morbid mental disorders are important individual risk factors to relapse. 

While preliminary, there is some preclinical evidence showing that treatment with CBD might promote resilience to developing cocaine and METH use disorders and might prevent relapse into drug use after a period of abstinence. The authors hypothesized that such protective actions of CBD on addiction to psychostimulant drugs may be related with the capacity of this cannabinoid compound to reverse the neurobehavioral alterations induced by these drugs that enhance the vulnerability to relapse. 

### 3.1. CBD Could Prevent Neuroadaptations Induced by Psychostimulant Drugs

There is some evidence that CBD modulates various neuronal circuits involved in drug addiction. Firstly, CBD interferes with the brain reward mechanisms, since high doses (10 and 20 mg/kg) significantly elevated the threshold frequency required for medial forebrain bundle ICSS [36]. This could indicate that CBD has an anti-reward effect. However, in the same study, a lower dose of CBD (5 mg/kg) did not modify the acute reinforcing properties of cocaine, although it was effective to reduce those of morphine [36]. Secondly, CBD could attenuate the psychostimulant drug-induced dysregulation of the dopaminergic mesolimbic system. It has been demonstrated that the intrahypothalamic administration of CBD increased levels of DA and adenosine in the NAcc [65,66] while systemic administration of CBD increased c-fos expression in this structure but not in the dorsal striatum [67]. A recent study also demonstrated that the administration of CBD significantly reduced relative gene expression of tyrosine hydroxylase in the ventral tegmental area (VTA) [68]. Furthermore, CBD attenuated the alterations in the mesolimbic circuitry induced by amphetamine [56]. Following an amphetamine challenge, amphetamine-sensitised rats displayed an increase in the firing frequency of DA neurons of the ventral tegmental area (as well as psychomotor sensitization and a deficit of pre-pulse inhibition). The administration of CBD into the NAcc reversed all these physiological and behavioural effects [56,69,70]. Electrophysiological recordings showed that CBD-treated rats displayed a decreased VTA DA neuronal firing frequency and bursting levels after the amphetamine challenge [56]. Moreover, CBD also attenuated the oxidative stress induced by amphetamine in the NAcc [71]. In addition to the evidence with psychostimulant drugs, Ren et al. [72] demonstrated that CBD exposure attenuated the alterations in the endocannabinoid and glutamatergic neurotransmission in the NAcc that were associated with cue-induced reinstatement of heroin self-administration. In rats exposed to heroin, CB1R mRNA expression was increased in the NAcc, this effect being reduced by CBD (even two weeks after its administration). On the other hand, cue-induced reinstatement was accompanied by a marked reduction of AMPA GluR1 protein expression in the NAcc, an effect that was normalised 24 h after CBD. In addition, in that study, a single injection of CBD (administered 24 h before the reinstatement session) attenuated cue-induced reinstatement of heroin seeking. This inhibitory effect of CBD on reinstatement was observed even two weeks after the last injection of CBD (5 mg/kg daily over three days) [72].

Taken together, these results suggest that the capacity of CBD to reverse the increase in the activity of the mesolimbic DA reward system induced by the exposure to drugs of abuse may be one of the most important mechanisms underlying its usefulness against the dependence to psychostimulants and other drugs of abuse.

### 3.2. CBD Could Reverse the Behavioural Effects of Psychostimulant Drugs through the Action on Different Neurotransmitter Systems and Intracellular Signalling Pathways

**Figure 1:**
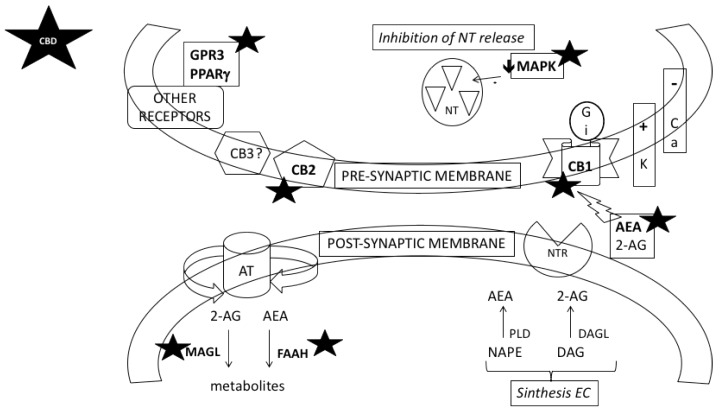
Components of cannabinoid synapsis modulated by CBD (★). The two main endocannabinoids (EC), anandamide (AEA) and 2-arachidonylglicerol (2-AG), are synthesized in the post-synaptic nerve terminal. AEA is produced by hydrolysis of N-arachidonyl phosphatidylethanolamine (NAPE) which is catalized by the enzyme phospholipase D (PLD). 2-AG is produced by the metabolism of diacylglicerol (DAG) by specific diacylglicerol lipases (DAGL). These EC act mainly through cannabinoid CB1 and CB2 receptors, although other receptors have been identified as targets of EC including a non-cloned CB3 receptor, GPR3, and the peroxisome proliferator activated-receptor gamma (PPARg). EC function as retrograde signalling molecules that inhibit the release of classical anterograde neurotransmitters (NT) by presynaptic terminals and binding with their receptor (NT receptor, NTR). After the activation of presynaptic CB1 receptors (by AEA or 2-AG), different signal transduction mechanisms are stimulated via G inhibitory proteins. EC reduce activity of protein kinases, as mitogen-activated protein kinase (MAPK), modulate ion channels (stimulation of potassium and inhibition of calcium channels) and inhibit NT release. The activity of endocannabinoids is limited by a transporter (AT) that reuptakes AEA and 2-AG into the post-synaptic cell. AEA is degraded by the enzyme fatty acid amidohydrolase (FAAH) and 2-AG is degraded by the enzime monoacylglicerol lipase (MAGL). CBD has been demonstrated to modulate anandamide signalling, CB1 and CB2 receptors, GPR3 and PPARg receptors and the activity of the enzymes FAAH and MAGL.

CBD is a very low-affinity CB1 ligand that can behave like an inverse agonist of CB1 receptors through an indirect action [11,73]. For this reason, CBD could produce the therapeutic actions of SR141716 (rimonabant) without inducing unwanted side effects. In support of the role of CB1 receptors in the effects of CBD, it has been reported that the inhibition of the rewarding effects of cocaine in the CPP and self-administration paradigms induced by CBD was parallel to the increase in the expression of these receptors in the hippocampus [54]. In another recent work, the administration of CBD reduced the gene expression of the CB1 receptor in the NAcc but increased that of the CB2 receptor [68]. Other studies have indicated that the effects of CBD are mediated by other non-cannabinoid receptors. For example, the protective effect of CBD on cocaine-induced seizures is not reversed by either the CB1 or CB2 receptor antagonists (AM251 and AM630, respectively), suggesting that alternative mechanisms are involved [52]. As stated by Adamczyk et al. [74], the catabolic enzymes FAAH and MAGL could also have a role in the effects of CBD. Figure 1 shows the components of cannabinoid synapsis modulated by CBD.

Functional interactions of CBD with the serotonin 5-HT1A receptor system may also be responsible for its effects [70]. CBD acts as an allosteric modulator of 5-HT receptors [10] and has agonistic properties at 5-HT1A receptors [20,21,75]. Psychostimulant drugs increase serotonin and the 5-HT1A receptors play an opposite role in the addiction to these drugs in function of their pre- or post-synaptic localisation [76]. It is not clear whether the action of CBD on 5-HT1A receptors may contribute to its effects on cocaine and METH reward. Buspirone, a 5HT1A agonist, prevented cue-induced reinstatement of cocaine and METH self-administration. However, these effects may also be due to the actions of buspirone on other receptors given its complex neuropharmacology [77]. Other studies have demonstrated that selective 5HT1A agonists reduced the hyperactivity and the psychomotor sensitization induced by METH [78,79]. The authors can hypothesise that CBD could prevent the rewarding effects of psychostimulant drugs through an agonistic action on post-synaptic 5-HT1A receptors, whose activation predominantly inhibits several addiction-related behaviours [80]. In addition, the action of CBD as a partial agonist of DA D2 receptors [26] could also explain its effects on psychostimulant reward, since other D2 partial agonists, such as aripiprazole or terguride, attenuated the self-administration of cocaine [81,82,83,84], amphetamine [85] and METH [86].

CBD also induces a non-competitive inhibition of the α7-nicotinic acetylcholine receptors [24]. This effect of CBD might also contribute to its influence on the rewarding effects of psychostimulant drugs, since it has been reported that the administration of methyllycaconitine (a selective antagonist of α7-nicotinic receptors) attenuated the reinforcing effects of cocaine in the ICSS paradigm [87], prevented the sensitization of the increase in extracellular accumbal DA levels induced by cocaine [88] and protected against neurotoxicity and glial activation induced by METH [89].

The actions of CBD on other receptors can also mediate its effects on psychostimulant addiction-related behaviours. These neuropharmacological actions include: The allosteric modulation of mu- and delta-opioid receptors [25], since CBD treatment reduced the gene expression of mu-opioid receptor in the NAcc [68]; the reduction of GluA1/2 AMPA subunit receptor ratio in the striatum [54], since cocaine increased glutamate release while CBD reduced it in hippocampal synaptosomes [52]; the effects of CBD on GPR3 [33], since GPR3 modulates the early phases of cocaine reinforcement [90]; and, finally, the action of CBD as an agonist of the peroxisome proliferator-activated receptor (PPAR) gamma [91,92], since the PPAR gamma agonist pioglitazone relieved the expression of behavioural sensitization to METH in mice [93] and reduced craving in cocaine-dependent individuals seeking treatment [94].

At the molecular level, modification in kinases activity may be one of the mechanisms underlying the actions of CBD on the effects induced by psychostimulant drugs. The protective effect of CBD against cocaine-induced seizures was reversed by rapamycin, an inhibitor of the mammalian target of rapamycin (mTOR) intracellular pathway. The authors of that study suggested that this preventive effect of CBD “possibly occur[s] through activation of mTOR with subsequent reduction in glutamate release” [52]. In another study, the inhibition of the mTOR/p70S6K pathway blocked the effects of CBD on amphetamine-induced psychomotor sensitization and pre-pulse inhibition deficit. In addition, amphetamine sensitised-rats treated with CBD showed changes in the expression of proteins of the Wnt (GSK-3, Akt, -catenin) and mTORC1 (mTOR, p70S6K) signal transduction pathways in the NAcc. In particular, amphetamine sensitised-rats treated with CBD (100 ng/0.5 µL) showed a decrease in phosphorylated-GSK-3β and phosphorylated-Akt, but an increased in phosphorylated-mTOR and phosphorylated-p70S6K. Further, the same changes were induced in the ratio of phosphorylated levels to the total levels of these proteins in the NAcc [56]. A recent study also demonstrated that mice self-administering cocaine and treated with CBD showed an upregulation of MAPK-CREB signalling (increased phosphorylation of ERK1/2 and CREB proteins) and elevated BDNF expression in the hippocampus [54].

### 3.3. CBD could Reverse the Alterations in the Immune System and the Neuroinflammation Induced by Psychostimulant Drugs

Preclinical studies have demonstrated that drugs of abuse induce neuroinflammatory effects and disrupt glutamate homeostasis through their interaction with microglia and astrocytes. Due to this, glial modulators, anti-oxidants and anti-inflammatory drugs show therapeutic potential in animal models of substance use disorders [95,96]. Neuroinflammation provoked by chronic exposure to cocaine contributed to cocaine seeking as is indicated by the fact that the antagonism of Toll-Like Receptor 4 (TLR4) in the VTA reduced priming-induced reinstatement of cocaine self-administration [97]. Similarly, an enhancement of cytokines (tumor necrosis factor α (TNF-α) interleukin-1β (IL-1β), IL-6 and IL-10) in the prefrontal cortex and hippocampus triggers the reinstatement of METH-induced CPP [98].

In models of ischemia, neurodegenerative disorders (Alzheimer’s disease and multiple sclerosis), sciatic nerve injury, epilepsy and schizophrenia, CBD reduces proinflammatory effects and the increased activity in astrocytes that characterises these disorders [99]. However, the studies on the actions of CBD in the neuroinflammation induced by the drugs of abuse are scarce. With respect to psychostimulant drugs, Karimi-Haghighi et al. [98] have reported that the icv administration of CBD (10 μg/5 μL) prevented reinstatement of METH-induced CPP through a change of gene expression of cytokines. After extinction of CPP, priming-induced reinstatement of METH CPP enhanced mRNA expression of TNF-α and IL-10 in the prefrontal cortex (PFC) and the hippocampus. Stress- and priming-induced reinstatement of CPP increased the expression of TNF-α and IL-1β in the PFC, and of TNF-α, IL-6 and IL-10 in the hippocampus. The administration of CBD prevented the increases of cytokines in the PFC (IL-1β, IL-6, and IL-10) and the hippocampus (TNF-α, IL-1β, and IL-6). In stressed rats, CBD also diminished IL-10 in the PFC, although it increased the expression of cytokines (TNF-α, IL-1β, IL-6, and IL-10) in the hippocampus [98]. 

Other studies have also demonstrated that CBD exerted a neuroprotective effect against the adverse consequences of alcohol in the hippocampus [46] and reduced the microglia reactivity induced by nicotine withdrawal. This attenuated the increase in the levels of neuroinflammatory markers IL1β in the hippocampus and IFNγ in the PFC [100]. According to these data, it could be hypothesized that CBD may prevent the microglial reactivity and neuroinflammation induced by cocaine.

### 3.4. CBD could Erase the Aberrant Drug-Related Memories Affecting the Reconsolidation Process

The release of DA induced by cocaine, METH and other drugs of abuse, in addition to underlying their rewarding effects, strengthens the association between the pleasant effects of these drugs and the contextual cues present when such effects are experienced by the subjects. Thus, drugs of abuse induce the creation of aberrant memories related to the drug experience. Even after long-term abstinence, exposure to drug-associated environmental cues activates the recalling of drug-related memories, thereby inducing craving and frequently leading to a relapse into drug use. Several studies have demonstrated that the manipulation of the reconsolidation process may be therapeutically used to erase pathological emotional memories such as fear- and drug-related memories [101,102]. In the experiments of reconsolidation, drug-related memories are actively reactivated exposing the subjects to environmental cues previously associated with drug consumption. After reactivation, the administration of a pharmacological or behavioural treatment (extinction) in the window of reconsolidation is used to substitute the original drug memory by a new non-drug memory [103]. A preclinical study has demonstrated that the administration of CBD just after the reactivation of a previously acquired cocaine CPP disrupted the reconsolidation of this CPP as evidenced by the fact that the preference was not reinstated by drug priming or stress exposure. As the authors of that study indicated, these results suggest a “therapeutic potential (of CBD) to attenuate contextual memories associated with drugs of abuse and consequently to reduce the risk of relapse” [60]. 

Different studies have confirmed that CBD regulates the processing of emotional memories [101,102]. However, the effects of this compound on aversive fear memories have been more broadly studied than its effects on drug-related memories in preclinical studies and in humans [101,104,105,106,107,108]. These studies have allowed the identification of neurobehavioral mechanisms involved in the CBD-induced reduction of emotional memory, which may be achieved through the facilitation of the extinction and/or the disruption of reconsolidation [101,104]. Both processes seem to be mediated by the activation of the endocannabinoid system, which is induced by the stimulation of CB1/CB2 receptors by anandamide in the PFC, amygdala, hippocampus, and/or NAcc [102]. Thus, it can be hypothesised that the blockade of reconsolidation of drug memories may be due to an indirect effect of CBD on endocannabinoid transmission via anandamide-mediated activation of CB1 and/or CB2 receptors. The erasure of a drug-related memory may be a mechanism underlying the therapeutic potential of CBD in psychostimulant addiction.

### 3.5. CBD could Reverse the Cognitive Deficits Induced by Psychostimulant Drugs

Chronic consumption of psychostimulant drugs is associated with cognitive deficits in attention, memory and executive functions [109,110]. The treatment and improvement in these cognitive deficits could prevent drug relapse [111,112]. CBD has shown pro-cognitive effects in preclinical studies. In mice, the pre-treatment with an intermediate dose of CBD (20 mg/kg) for 10 days facilitated the novel object recognition task and increased learning markers, such as BDNF expression and neural progenitor proliferation in the hippocampus [54]. CBD blunted the cognitive impairment induced by THC in an adenosine A2A receptor-dependent manner [113] and abolished the memory impairment in the object recognition task induced by nicotine withdrawal (precipitated by the administration of the nicotinic antagonist mecamylamine) as well as the neuroinflammatory response and the impairment in neurogenesis associated with these cognitive deficits (as previously commented) [100].

An open-label clinical trial with frequent cannabis users treated with 200 mg of daily oral CBD treatment for 10 weeks, while continuing to use cannabis, reported improvements in attention, verbal learning, and memory [114]. In cigarette smokers, acute nicotine abstinence also produced deficits in verbal and spatial working memory and impulsivity (go/no-go, delay discounting, prose recall and N-back tasks). However, conversely to that observed in animal models, such deficits were not reversed by the administration of CBD (800 mg oral) and this compound even increased commission errors on the go/no-go task in comparison to the placebo treatment [115]. Future preclinical and clinical studies should evaluate whether CBD could attenuate the cognitive deficits induced by chronic exposure to cocaine and METH.

### 3.6. CBD could Alleviate the Mental Disorders Comorbid with Psychostimulants Abuse

CBD has been shown to have a potential therapeutic value for a wide range of disorders, including stress, anxiety, psychosis, depression, as well as for problems related to sleep and appetite [16,41,114,116]. The capacity of CBD to alleviate mental symptomatology may mediate its benefits to treat psychostimulant addiction.

Stress is an environmental variable clearly associated with initiation, maintenance and relapse into drug abuse and is a challenge for the treatment of substance use disorders. According to Greenwald [117], CBD can be considered an emerging pharmacotherapy with the potential for reducing stress-potentiated seeking and consumption of several drugs of abuse, including cocaine. In laboratory rodents, acute and repeated treatment with CBD produced anxiolytic effects in the elevated plus-maze [54,57,59] and other behavioural paradigms [16]. It also reduced the anxiogenic effects of chronic unpredictable stress in mice [14]. Currently available data in humans also support the therapeutic potential of CBD for the treatment of anxiety disorders such as panic disorder, generalised and social anxiety disorder, obsessive-compulsive disorder and post-traumatic stress disorder [104,118,119,120]. The therapeutic effect of CBD may be related with the attenuation of aversive/traumatic memories by facilitating its extinction. As previously commented, the indirect activation of CB1 receptors induced by CBD during the formation of a traumatic memory or its reactivation can facilitate the subsequent extinction of this memory [121,122]. The agonism of the 5-HT1A receptors also seems to contribute to the reduction of anxiety induced by CBD [16,120]. As panic and post-traumatic stress disorders have been associated with drug addiction, it may be expected that the improvement of these stress-related psychiatric conditions could consequently reduce the abuse of psychostimulant drugs. Thus, studies with laboratory animals and human beings showed an anxiolytic-like effect of CBD, suggesting that this compound could be a promising drug for the treatment of stress-related disorders. However, more preclinical and clinical research need to be performed in order to confirm the mechanisms underlying the therapeutic potential of CBD.

Impulsive and compulsive behaviour have also been related with an enhanced propensity to develop substance-related disorders. Using a delay-discounting task, it has been observed that CBD prevented the development of high impulsivity in rats with a history of alcohol dependence [59]. However, CBD did not modify impulsivity during abstinence in tobacco-dependent subjects [115]. With respect to compulsive behaviour, CBD inhibited such behaviour in the marble burying test in mice [123] but did not have any effects in the schedule-induced polidipsia model [124]. 

There is a clear association between psychostimulant abuse and psychosis [125]. CBD has antipsychotic effects [126] and may reduce the affective and cognitive deficits associated with schizophrenia, mainly through the facilitation of endocannabinoid signalling and CB1 receptor antagonism [120], the reduction of glial reactivity [127] and the normalisation of molecular and neuronal changes that take place in the mesolimbic system [70]. Hudson et al. [128] also hypothesised that CBD could prevent the acquisition of emotionally irrelevant memories and reverse schizophrenia-related pathology, since the stimulation of CB1 receptors in the ventral hippocampus potentiates the formation of affective memories. To test the antipsychotic efficacy of CBD, studies with rodents and non-human primates have used the pre-pulse inhibition (PPI) of acoustic startle reflex, a paradigm that models sensorimotor gating deficits observed in several neuropsychiatric disorders [129]. CBD reversed the PPI deficits induced by N-methyl d-aspartate receptor antagonists [129,130]. Similarly, the deficit in the PPI induced by the repeated exposure to amphetamine is reduced by the administration of CBD into the NAcc, an effect that is blocked by the inhibition of the mTOR/p70S6K pathway in this brain area [56]. Taken together, these results suggest the usefulness of CBD in the treatment of neurologic disorders that present alterations in the sensorimotor system, such as schizophrenia and drug addiction.

Finally, there is an association between drug addiction and depression and it could be expected that the improvement of depressive symptoms, such as helplessness and anhedonia, may be accompanied by a reduction of drug use. In experimental rodents, CBD had anti-depressive effects in the forced swim test [131,132,133,134,135], tail suspension test [136,137,138,139], sucrose preference test [75], saccharin preference test [139] and the learned helplessness paradigm [133]. Indirect activation of CB1 receptors in the ventromedial PFC [134], stimulation of serotonin [132] and 5-HT1A receptors [16,75,135,140], enhanced cortical 5-HT/glutamate neurotransmission [75] and increased BDNF- TrkB-mTOR signalling in the amygdala, medial PFC and the hippocampus [133,134,136] have been involved in the antidepressant effects of CBD. Thus far, there is no evidence for the usefulness of CBD in human depression.

## 4. Conclusions

A limited number of preclinical studies indicate that CBD could have therapeutic properties on cocaine and METH addiction and some preliminary data suggest that CBD may be beneficial in cocaine-crack addiction in humans. CBD has shown promising results in reducing the inflammation and seizures induced by cocaine [51,52] and in several preclinical models of addiction to amphetamine [38], cocaine [38,54,59] and METH [56,61,98]. Importantly, a brief treatment of CBD induces a long-lasting prevention of reinstatement of cocaine and METH seeking behaviours. However, in other studies, CBD treatment had a minimal effect on cocaine reinstatement [54,57]. These controversial results indicate that the efficacy of CBD may be dependent on a range of factors, including the dose and schedule of administration of this compound (i.e., acute or repeated; before, co-administered with, or after the psychostimulant drug), the type of substance of abuse under study (cocaine, amphetamine or METH), the paradigm used to evaluate psychostimulant addiction (ICSS, self-administration, conditioned place preference or behavioural sensitization) and the process studied (acquisition, extinction, reinstatement or reconsolidation). In the self-administration paradigm, CBD showed higher efficacy to block the reinstatement than the acquisition or maintenance of self-administration [54]. Furthermore, in the CPP paradigm, CBD was more effective to accelerate extinction [38] or to impair reconsolidation [60] than to block the acquisition or expression of the place conditioning induced by cocaine or amphetamines [38]. In addition, context-induced reinstatement of cocaine seeking was prevented by CBD [59] while this compound did not affect priming-induced reinstatement [54]. These results may reflect a predominant role of CBD in attenuating drug-related memory, without altering the reinforcing or rewarding properties of cocaine. The evidence of CBD’s role in regulating emotional memory is further supported by the blockade of stress-induced reinstatement of cocaine seeking [59]. CBD was also able to block priming-induced reinstatement of METH seeking but this effect was observed only with high doses [58] or icv administration [61,98]. In comparison to other drugs of abuse, CBD seems to have a relatively weaker efficacy in disrupting the rewarding and reinstating effects of psychostimulant drugs. For example, on the ICSS paradigm, CBD did not alter the effects of cocaine with an effective dose to inhibit the effects of morphine [36]. Similarly, CBD prevented cue-induced reinstatement of heroin [72], but not cocaine self-administration [57].

**Figure 2:**
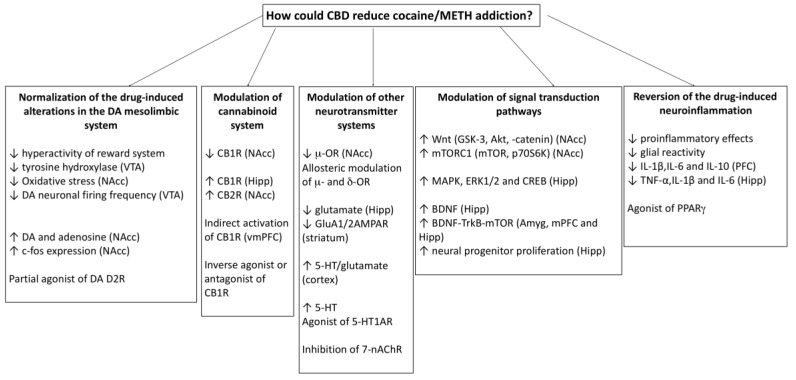
Hypothetical mechanisms involved in the effects of CBD on cocaine/METH addiction. The involvement of these processes is supported by the results of preclinical studies. VTA: ventral tegmental area; DA: dopamine; NAcc: nucleus accumbens; DA D2: dopamine D2 receptors; CB1R; cannabinoid receptors type 1; Hipp: hippocampus; CB2R; cannabinoid receptors type 2; vmPFC: ventromedial prefrontal cortex; m-OR: mu opioid receptors; d-OR: delta opioid receptors; 5-HT: serotonin; 5-HT1AR: type 1A serotonin receptors; 7-nAChR: nicotinic acethylcholine receptors type 7; GSK3: glycogen synthase kinase-3; Akt: protein kinase B; mTORC1: mammalian target of rapamycin complex 1; MAPK, mitogen-activated protein kinase; ERK1/2: Extracellular signal-regulated kinases type 1 and type 2; BDNF: brain derived neurotrophic factor; TrkB: Tropomyosin receptor kinase B; Amyg: amygdala; mPFC: medial prefrontal cortex; IL: interleukine; TNF- α: tumor necrosis factor alpha; PPARg: Peroxisome proliferator-activated receptor gamma.

Drug addiction is characterized by the compulsive desire to use drugs and a loss of control over consumption. For this reason, in the preclinical field, the effects of CBD need to be evaluated in experimental models of drug-escalation, which translate more readily to drug abusers searching treatment, who have initiated and progressed their drug use until developing a compulsive pattern of consumption. Future preclinical studies should evaluate the effects of CBD on priming- and stress-induced reinstatement of cocaine CPP as well as on the negative consequences of withdrawal after repeated exposure to cocaine and METH. Some of these issues are currently being researched in the authors’ laboratory. Further studying of the mechanisms of action underlying CBD’s therapeutic potential for psychostimulant addiction will be also necessary. The reversion of psychostimulant-induced alterations in the DA mesolimbic system and the minimization of inflammatory injury promoted by cocaine or METH seem to be important mechanisms. Moreover, the protracted behavioural effects of CBD suggest that this compound has a long-term impact on synaptic plasticity, which is mediated by the endocannabinoid system [141] and altered by addictive drugs [142]. According to Gerdeman et al. [53], the endocannabinoid-mediated synaptic plasticity “may act specifically within drug-paired environments to maintain cocaine-directed behavioural responses”. Figure 2 shows the hypothetical mechanisms (based on the results of preclinical studies) involved in the effects of CBD on cocaine/METH addiction. 

The FDA has approved a purified form of CBD (Epidiolex) to treat rare, severe forms of epilepsy. However, there are no other FDA-approved drug products that contain CBD for the treatment of any other diseases. The cause of this lies in the FDA’s priority to protect and promote public health and, consequently, this Agency is committed to science-based decision-making. The FDA recognizes CBD’s potential benefits, but also maintains that questions remain regarding its safety, including the potential for liver injury (identified during the review of the marketing application for Epidiolex). Currently, CBD is the subject of substantial clinical research on its potential medical uses, including the treatment of addiction to drugs of abuse, but there remain many open questions that need to be considered before CBD may be more widely available. For example, what is the maximal dose of CBD that is safe to consume daily? How does CBD’s safety depend on the method of administration? What are the risks of long-term exposure? Are there drug interactions? How does CBD impact special populations (such as adolescents or pregnant women) and people with chronic diseases? In addition to the safety issues, the effectiveness of CBD for treating addiction to psychostimulant drugs has not yet been proven. The paucity of preclinical findings and the lack of results derived from clinical trials greatly hinder the FDA’s approval of CBD to treat addiction to cocaine or METH. From the case of Epidiolex, the FDA only supports a drug’s approval after careful review of the evidence obtained in scientifically valid research and the conclusion that CBD is safe and effective for its intended use. Thus, a priority of research is to demonstrate the usefulness of CBD as a novel pharmacotherapy for psychostimulant dependence in clinical trials. These future clinical trials should include randomised controlled samples of individuals with the diagnosis of cocaine or METH dependence (without or with comorbid neuropsychiatric conditions) to investigate the specific effects of CBD on these disorders, the specific mechanism of action of CBD and the safe and ideal therapeutic doses of this compound. Biological measures must also be included in these clinical trials in order to correlate the therapeutic effects of CBD to changes in neurotransmitters, intracellular signalling, neuroinflammatory markers, and structural and functional cerebral changes [120]. It is needed to obtain clinical evidence for the safety and effectiveness of CBD through adequate and well-controlled clinical trials for the translation of CBD in the treatment of psychostimulant abuse and addiction.

In conclusion, there is currently limited evidence of the potential therapeutic benefits of CBD for cocaine and METH use disorders and commonly related adverse symptoms. A clear limitation of the literature is the paucity of human research and the lack of clinical trials. Given the absence of abuse liability of CBD and its general tolerability, this compound appears to be a promising candidate for pharmacotherapy of psychostimulant drug-use disorders. Further preclinical studies and future clinical trials are clearly necessary to fully evaluate the potential of CBD as an intervention for cocaine and METH addictive disorders. The validation of the safety and efficacy of CBD in reducing craving and relapse in preclinical and clinical trials will be necessary for the translation of research findings into clinical settings.
